# Src Kinases Are Required for a Balanced Production of IL-12/IL-23 in Human Dendritic Cells Activated by Toll-Like Receptor Agonists

**DOI:** 10.1371/journal.pone.0011491

**Published:** 2010-07-09

**Authors:** Mirela Kuka, Roberta Baronio, Sara Valentini, Elisabetta Monaci, Alessandro Muzzi, Susanna Aprea, Ennio De Gregorio, Ugo D'Oro

**Affiliations:** Novartis Vaccines, Siena, Italy; New York University, United States of America

## Abstract

**Background:**

Pathogen recognition by dendritic cells (DC) is crucial for the initiation of both innate and adaptive immune responses. Activation of Toll-like Receptors (TLRs) by microbial molecular patterns leads to the maturation of DC, which present the antigen and activate T cells in secondary lymphoid tissues. Cytokine production by DC is critical for shaping the adaptive immune response by regulating T helper cell differentiation. It was previously shown by our group that Src kinases play a key role in cytokines production during TLR4 activation in human DC.

**Principal Findings:**

In this work we investigated the role of Src kinases during different TLRs triggering in human monocyte-derived DC (MoDC). We found that Src family kinases are important for a balanced production of inflammatory cytokines by human MoDC upon stimulation of TLR3 and 8 with their respective agonists. Disruption of this equilibrium through pharmacological inhibition of Src kinases alters the DC maturation pattern. In particular, while expression of IL-12 and other inflammatory cytokines depend on Src kinases, the induction of IL-23 and co-stimulatory molecules do not. Accordingly, DC treated with Src inhibitors are not compromised in their ability to induce CD4 T cell proliferation and to promote the Th17 subset survival but are less efficient in inducing Th1 differentiation.

**Conclusions:**

We suggest that the pharmacological modulation of DC maturation has the potential to shape the quality of the adaptive immune response and could be exploited for the treatment of inflammation-related diseases.

## Introduction

The onset of adaptive immunity is initiated by the phagocytosis of pathogens or their products by antigen-presenting cells (APCs), which present the antigens in the form of a peptide-MHC complex displayed on their surface to naïve T cells thus triggering the T cell receptor (TCR) [1]. In addition to TCR engagement, the interaction of co-stimulatory molecules on the APCs with their respective receptors on the T cell is required for T cell activation and proliferation [2]–[4]. Cytokines secreted by the dendritic cells (DC) serve as the third signal in T cell activation and modulate T cell differentiation into specific functional subsets. For example, CD4+ T lymphocytes can polarize toward different T helper cell types upon their activation. More than 20 years ago a series of studies led to the formulation of the Th1/Th2 paradigm. Th1 cells produce IFNγ and facilitate the onset of response against intracellular pathogens, while Th2 cells secrete mainly IL-4 and mediate protection from extracellular microbial agents [5]–[7]. During these last years this paradigm was challenged by the discovery of a new subset of T helper cells, the Th17 cells. This subset is distinct from the classical Th1 and Th2 subsets, since these cells produce IL-17, a pleiotropic inflammatory cytokine involved in the induction of a variety of pro-inflammatory mediators and adhesion molecules on various cell types. Recent works suggest a key role for TGF-β, IL-1β and IL-6 in the lineage commitment of Th17 cells [8]–[12]. However, the maintenance and full effector functions of Th17 cells are strictly related to IL-23, a heterodimeric cytokine [13] characterized by one specific subunit (*IL23A*/p19) and a second component shared with IL-12 (*IL-12B*/p40). Both IL-23 and IL-12 are induced in DC upon stimulation with different microbial stimuli, but they seem to drive T cell polarization in different directions. While IL-12 has a central role in Th1 differentiation, IL-23 is essential for the development of the full effector functions of the Th17 subset [13]. It has been shown that Th17 cells are critical in triggering inflammatory responses and have a key role in autoimmune diseases, at least in the murine model [14]–[16]. Other recent publications showed that Th17 cells play an important role in the reduction of inflammation in *Helicobacter pylori*-induced gastritis [17]. However, the exact role of Th1/Th17 polarization in inflammation has not been clearly elucidated yet.

The encounter between naïve T cells and antigen-loaded mature DC in T cell areas of secondary lymphoid organs is the first stage of T cell-based responses, and implies that DC maturation is a crucial requirement for the initiation of adaptive immunity [1]. The DC maturation process consists of the up-regulation of co-stimulatory molecules, an increase in surface peptide-MHC complex half-life and the production of inflammatory cytokines, and is triggered by a broad range of pathogen-associated molecular patterns (PAMPs). Most of these microbial products act as stimuli for Toll-like receptors (TLRs), an evolutionary conserved family of type-1 transmembrane receptors, inducing the triggering of two main signaling pathways. The majority of TLRs are characterized by the MyD88-dependent signaling pathway, while TLR3 activation leads to the TRIF-dependent cascade. TLR4 is the only member of the family that makes use of both signaling pathways. Both pathways, however, lead to the activation of transcription factors which are involved in the induction of inflammatory cytokines [18], [19].

Some evidences in the literature suggest an involvement of src-family tyrosine kinases in the signaling pathway of TLRs [20] [21]–[25]. In particular, it was recently demonstrated that Src kinases have a role in cytokine production by human macrophages stimulated with TLR agonists [21]. Earlier our group showed a critical role for src-family kinases in TLR4 signaling leading to cytokine production in monocyte-derived DC (MoDC) stimulated by LPS [25]. PP1, a specific inhibitor of src-family tyrosine kinases, was shown to block cytokine production in DC stimulated by LPS without affecting the capacity of this pathogen component to up-regulate the expression of MHC and co-stimulatory molecules on the surface of these cells. As a consequence, DC were still capable of stimulating T cell proliferation but were much less efficient in inducing Th1 differentiation.

Here we investigated the role of src-family kinases in cytokine/chemokine induction by triggering of TLRs other than TLR4. In particular we focused on TLR3, characterized by the TRIF-mediated signaling pathway, and on TLR8, which like the majority of TLRs, makes use of the MyD88-dependent cascade. We found that the Src kinase inhibitor PP2 was able to impair cytokine production by MoDC upon activation of both TLRs without affecting up-regulation of co-stimulatory molecules. Microarray experiments were performed to identify gene expression profiles associated with this biological behavior in MoDC. We found that stimulation of PP2-treated MoDC resulted in normal IL-23 production associated to inhibition of IL-12, and this molecular signature of Src kinases inhibition translated into a functional effect on T cell activation.

## Results

### Src kinases inhibition impairs production of inflammatory cytokines without affecting up-regulation of co-stimulatory molecules

**Figure 1:**
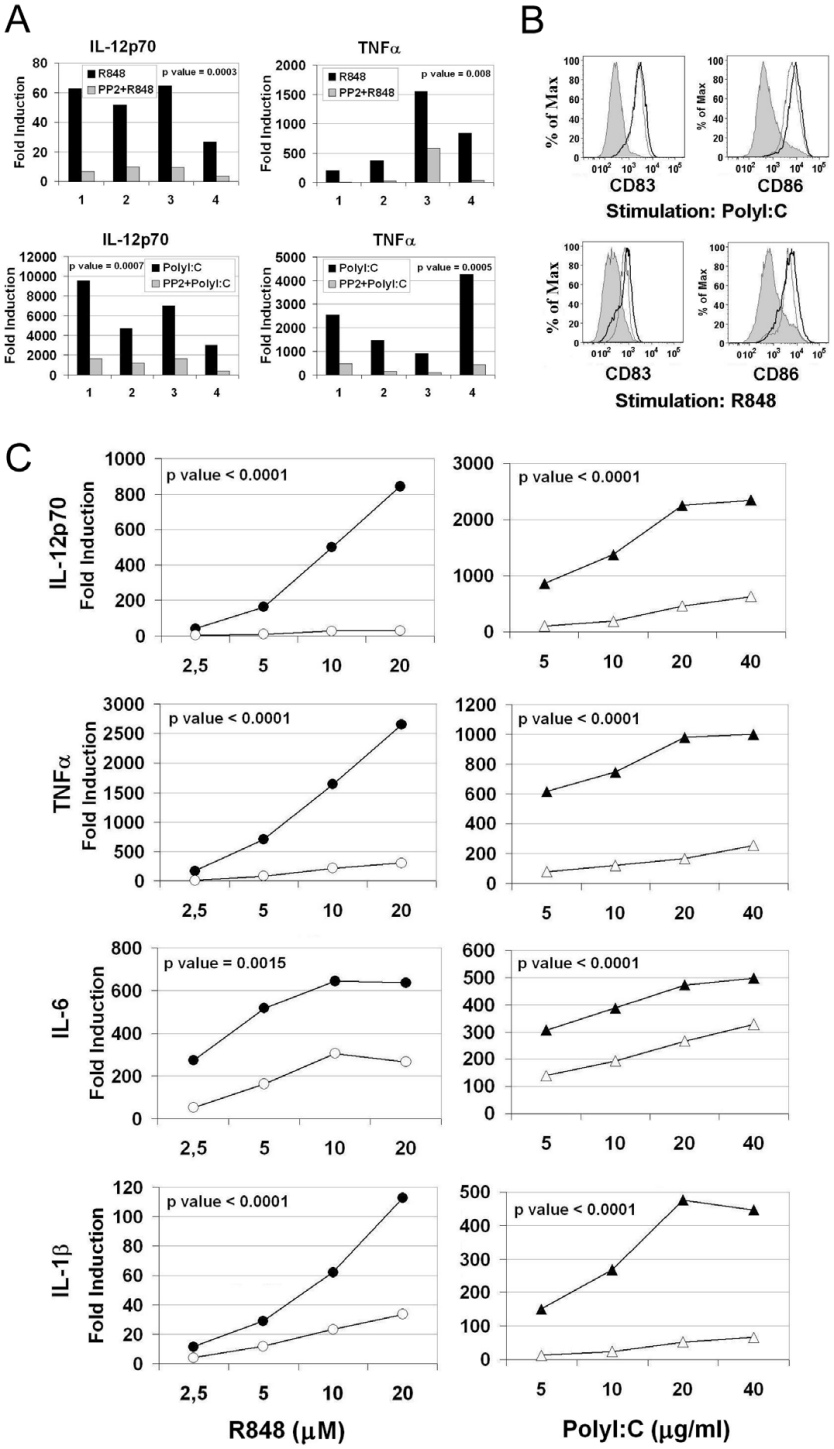
Src kinases inhibition results in uncoupling of cytokines production from up-regulation of co-stimulatory molecules. (A and B) Human MoDC were pretreated or not with PP2 (20 µM) and stimulated with PolyI∶C (20 µg/ml) or R848 (10 µM). (A) Cytokines released in the supernatants were detected after 24 hours of stimulation. Fold induction for the levels of each cytokine compared to unstimulated cells is plotted. Data from four independent experiments and the p values for the differences between the two experimental groups are shown (p value <0.05 is significant). (B) Surface expression of CD83 and CD86 was analyzed by flow cytometry after 36 hours of stimulation. Stimulated cells are represented with black line histogram, PP2-treated and stimulated cells are in grey line and untreated cells are in filled grey histogram. (C) MoDC were pretreated (open symbols) or not (filled symbols) with PP2 (20 µM) and stimulated with increasing concentrations of PolyI∶C and R848. Cytokines production was analyzed at 24 hours. Average fold induction of three independent experiments for the levels of each cytokine compared to unstimulated cells is plotted and the p values for the differences between the groups are shown (p value <0.05 is significant).

In a previous study [25] our group showed that Src kinases are involved in signaling cascades leading to cytokine production upon stimulation of TLR4 in human MoDC. Since TLR4 can signal through both the MyD88-dependent and -independent pathways, we asked if Src kinases play a role specifically in one of these cascades, or if they are involved in the signaling of all TLRs. In particular we focused on TLR3 for the TRIF-dependent and on TLR8 for the MyD88-dependent pathways, respectively. We used as agonists PolyI∶C for TLR3 and R848 for TLR8. Although R848 is known to activate both TLR7 and TLR8, only TLR8 is triggered in human MoDC since these cells do not respond to pure TLR7 agonists like Imiquimod. In accordance with previous studies, which showed that Src kinases are phosphorylated upon activation by TLR agonists [21], [24], [25], we found that Src phosphorylation at the activation site (Tyr 416) was increased when MoDC were stimulated with R848 and PolyI∶C (Figure S1). This phosphorylation was completely blocked by pretreatment of the cells with PP2, a Src kinases inhibitor, demonstrating the in-vivo specific activity of this compound (Figure S1). We then looked at functional effect of PP2 pretreatment on human MoDC stimulated with PolyI∶C or R848. Treatment of MoDC with PP2 inhibited the secretion of the inflammatory cytokines TNFα and IL-12p70 in response to both PolyI∶C and R848 as demonstrated by data from four independent experiments (Figure 1A), showing a statistical significant difference (p<0,05) between the two experimental groups. This impairment was associated to a normal expression and up-regulation of the co-stimulatory molecule CD86 and of the DC maturation marker CD83 (Figure 1B). In dose response experiments, pre-treatment with PP2 inhibited the secretion of IL-12p70, TNFα and other inflammatory cytokines such as IL-1β and IL-6, even at high concentrations of the agonists. Data from three independent experiments expressed as average fold induction with a statistical significant difference (p<0,05) between the two experimental groups are shown in Figure 1C. Moreover, since one of the outcomes of the TLR3-TRIF-dependent pathway is type I IFN production, we assessed the role of Src kinases on the induction of IFNβ. We found that IFNβ was induced only in PolyI∶C-stimulated cells, and this production was greatly impaired by Src kinases inhibition (Figure S2). These results confirmed that Src kinases play a key role in both TLR3 and TLR8 signaling pathways.

### Src kinases play a key role in the accumulation of c-Jun and IRF1

**Figure 2:**
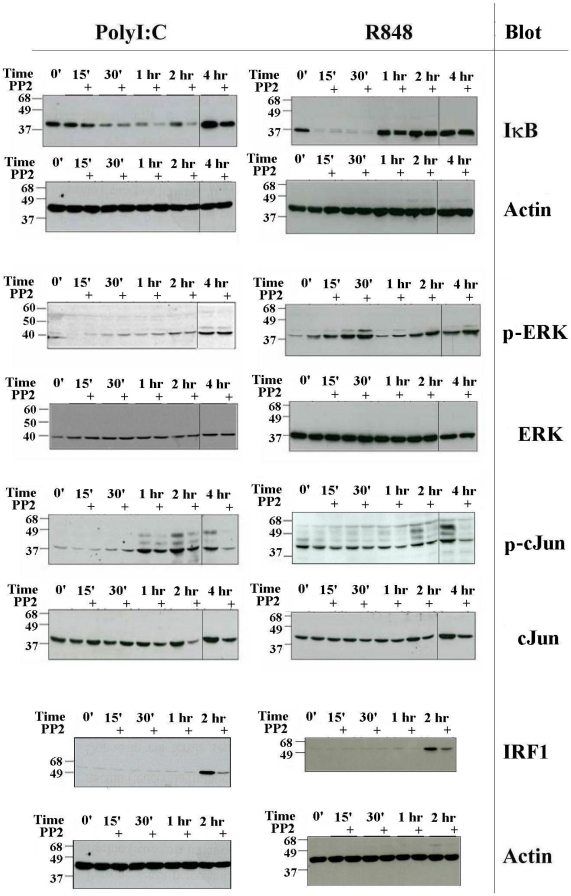
Src kinases are required for accumulation of c-Jun and IRF1. Human MoDC were pretreated or not with PP2 (20 µM) and stimulated with PolyI∶C (20 µg/ml) or R848 (10 µM) for the indicated time. IκB, phospho-ERK, phospho-cJun and IRF1 were detected by WB on total cell lysates. After stripping filters were re-blotted with antibodies to Actin, total ERK, total cJun and Actin, respectively. When samples from a single experiment were loaded on two different gels, a black line separates images obtained from the two separate blots. All data are representative of at least three experiments.

We then asked which is the role of Src-family kinases in the intracellular signaling pathways triggered by TLR3 and TLR7/8. Both the MyD88 and TRIF-dependent cascades are known to activate NF-κB, ERK and JNK pathways. As an indication of NF-κB activation after stimulation with R848 and PolyI∶C we monitored the degradation of IκB, and we found that it was not inhibited by treatment with PP2 (Figure 2 and Figure S3). On the contrary, in PP2-treated cells stimulated with PolyI∶C there was a delayed reappearance of IκB that could be explained by its sustained degradation at later time points. In fact no effect of PP2 on TLR3-induced transcription of IκB (NFKBIA gene) could be observed in microarray experiments (Table 1 and Table S1). This finding indicates that Src kinases do not have a negative role in the activation of the NF-κB pathway. We also found that PP2 did not inhibit ERK phosphorylation (Figure 2), or p38 phosphorylation (data not shown) induced by TLR3 or TLR8 activation. By contrast, pretreatment of MoDC with PP2 inhibited c-Jun phosphorylation and stabilization upon stimulation with R848 or PolyI∶C (Figure 2 and Figure S3A). The relevance of this observation is shown by the densitometric analysis of three independent Western blot experiments and the statistical significant difference (p<0,05) between the two experimental groups (Figure S3B). This demonstrates that Src kinases play an important role in modulating the activation of this transcription factor, which, as a component of the AP-1 complex, regulates the expression of the majority of cytokines upon TLR engagement. Induction of IRF1, another transcription factor known to control many cytokine genes, was also impaired by PP2 treatment (Figure 2 and Figure S3A). Again the densitometric analysis of three independent Western blot experiments and the statistical significant difference (p<0,05) between the two experimental groups (Figure S3B) demonstrate the relevance of src kinases for the induction of this transcription factor. Recently Zanoni et al [26] showed that stimulation of murine dendritic cells with LPS resulted in influx of extracellular calcium and activation of NFAT through a pathway that is CD14-dependent and TLR4-independent. We therefore tested if TLR3 or TLR8 stimulation could result in the activation of this pathway. However, we found that stimulation of human MoDC with PolyI∶C or R848 did not induce any calcium influx in these cells, confirming that TLRs do not directly activate an intracellular pathway that results in an increase of calcium levels (data not shown). All together these data suggest that Src kinases may play a key role in modulating cytokine induction in response to TLR agonists, through the regulation of key transcription factors.

**Table 1:** Gene expression profiles upon Src kinases inhibition.

	R848		PolyI∶C	
Gene Symbol	Fold change	Effect of PP2 (%)	Fold change	Effect of PP2 (%)
**Transcription activity (validated genes)**				
IRF8	4,8	−80,25	5,24	−64,05
NFKBIA			4,65	9,8
REL	5,72	26,55		
**Cytokines (validated genes)**				
IL12B	91,06	−85,66	45,06	−81,13
IL8	33,25	−82,69	11,81	−65,82
IL6	65,90	−73,79	88,99	−53,31
TNF	17,01	−66,29	19,07	−63,49
TNF	20,93	−34,13	26,54	−21,29
IL23A	41,41	101,86	18,35	6,13
**Other Cytokines**				
CCL1	8,53	−88,53	14,43	−93,93
IL1A	53,18	−92,43	86,91	−84,43
CSF2	10,97	−77,38	7,20	−83,72
CCL20	69,15	−77,37	57,19	−70,98
CXCL2	12,91	−41,72	16,90	−62,35
CXCL3	20,64	−39,57	24,26	−61,42
CXCL3	15,03	−42,76	16,44	−60,23
CXCL2	16,33	−42,23	27,82	−56,26
IL7R	6,83	−49,28	5,21	−48,97
CXCL1	83,24	−20,43	109,78	−45,66
IL10	9,68	−83,11	3,66	−36,78
IL15RA	10,64	−43,74	18,64	−20,84
CCL5	13,92	−45,39	27,91	−19,20
CLCF1	14,92	3,04	14,50	−18,40
IL1B	12,07	−14,90	40,98	−9,67
CCL4	6,37	−27,14	5,71	1,98
IL1F9	27,77	−93,87		
IL19	12,45	−92,34		
CXCL9			4,03	−21,66
CXCL11			5,28	−31,80
CXCL11			26,54	−59,31
IL18RAP			5,00	−59,83
IL28A			44,15	−61,07
IL28B			188,72	−73,24
CCL3L3			52,43	−74,54
CCRL2			153,81	−76,58
CCL3L3			158,11	−77,24
IFNB1			58,28	−82,31

Cytokine and transcription factor genes whose expression is up-regulated upon stimulation with R848 or PolyI∶C. Expression of some genes was validated by qRT-PCR. Fold induction upon TLR stimulation and the effect of PP2 for each stimulus is indicated. Blank spaces indicate that genes were up-regulated less than 4-fold upon stimulation. Some genes are indicated twice because two reporters were present in the array.

### Src kinases inhibition differentially affects gene expression profiles in stimulated MoDC

To further study the role of Src kinases in both the MyD88 and TRIF-dependent pathways, we performed a transcriptional analysis of cells stimulated with TLR3 and TLR8 agonists in the presence or in the absence of PP2. First, the transcriptome of MoDC stimulated for 4 hours with PolyI∶C or R848 was compared to the transcriptome of non-stimulated cells to identify all TLR3 and TLR8 responsive genes, respectively. By using a threshold of 4-fold up-regulation with a pvalue≤0.05 across three different MoDC donors we identified 145 TLR3 (Table S1) and 151 TLR8 (Table S2) dependent reporters. In order to evaluate the impact of PP2 treatment on these genes a second microarray experiment was performed using MoDC from the same donors. In this experiment the transcriptome of MoDC pre-treated with PP2 and stimulated with PolyI∶C or R848 for 4 hrs was compared with the transcriptome of corresponding MoDC stimulated in the absence of PP2.

**Figure 3:**
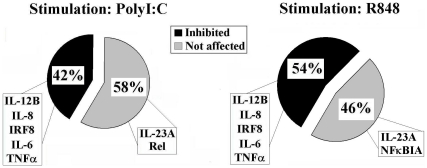
Graphical representation of gene expression modulation by Src kinases inhibition. Human MoDC were pretreated or not with PP2 (20 µM), stimulated with PolyI∶C (20 µg/ml) or R848 (10 µM) for 4 hours, and gene expression was assessed by Microarrays. Inhibition of gene expression by PP2 treatment was analyzed on genes which were up-regulated at least 4-fold upon stimulation, with a p value <0.05.

Approximately 42% of the Poly∶IC up-regulated genes and 54% of the genes up-regulated by R848, were inhibited more than 50% by PP2 (Figure 3). Among these inhibited genes we found cytokine-encoding genes like *IL-12B, TNF*α, *IL-6* and the transcription factor *IRF8* (Table 1). Consistently with western blot experiments, among the genes that were not affected by treatment with PP2 we found genes that are regulated by the NF-κB pathway, such as the NF-κB inhibitor and other members of the NF-κB family. Therefore this last finding strengthens our hypothesis that Src kinases are not involved in the NF-κB family pathways. Unexpectedly, transcription of the IL-1B gene was not dramatically impaired in PP2 treated cells, although the release of this cytokine upon TLR stimulation was regulated by Src kinases (Figure 1). These data suggest that Src kinases can modulate IL-1 production by a post-transcriptional mechanism.

IL-23 and IL-12 are two homologous cytokines characterized by a common p40 chain (IL-12B) and another chain specific for each cytokine (IL-12A or IL-23A). We found that the gene encoding the alpha subunit of the cytokine IL-23 *(IL-23A)* was not inhibited following pharmacological blockade of Src kinases in MoDC stimulated with either PolyI∶C or R848 (Table 1). Surprisingly, pre-treatment with PP2 resulted in an even higher up-regulation of *IL-23A* mRNA by R848 (2-fold more compared to R848 stimulated cells). In contrast, the induction of *IL-12B* by either PolyI∶C or R848 was inhibited by PP2 more than 80% (Table 1). No conclusion on *IL-12A* transcription could be obtained from microarray data due to a high variability of the results among different donors.

**Figure 4:**
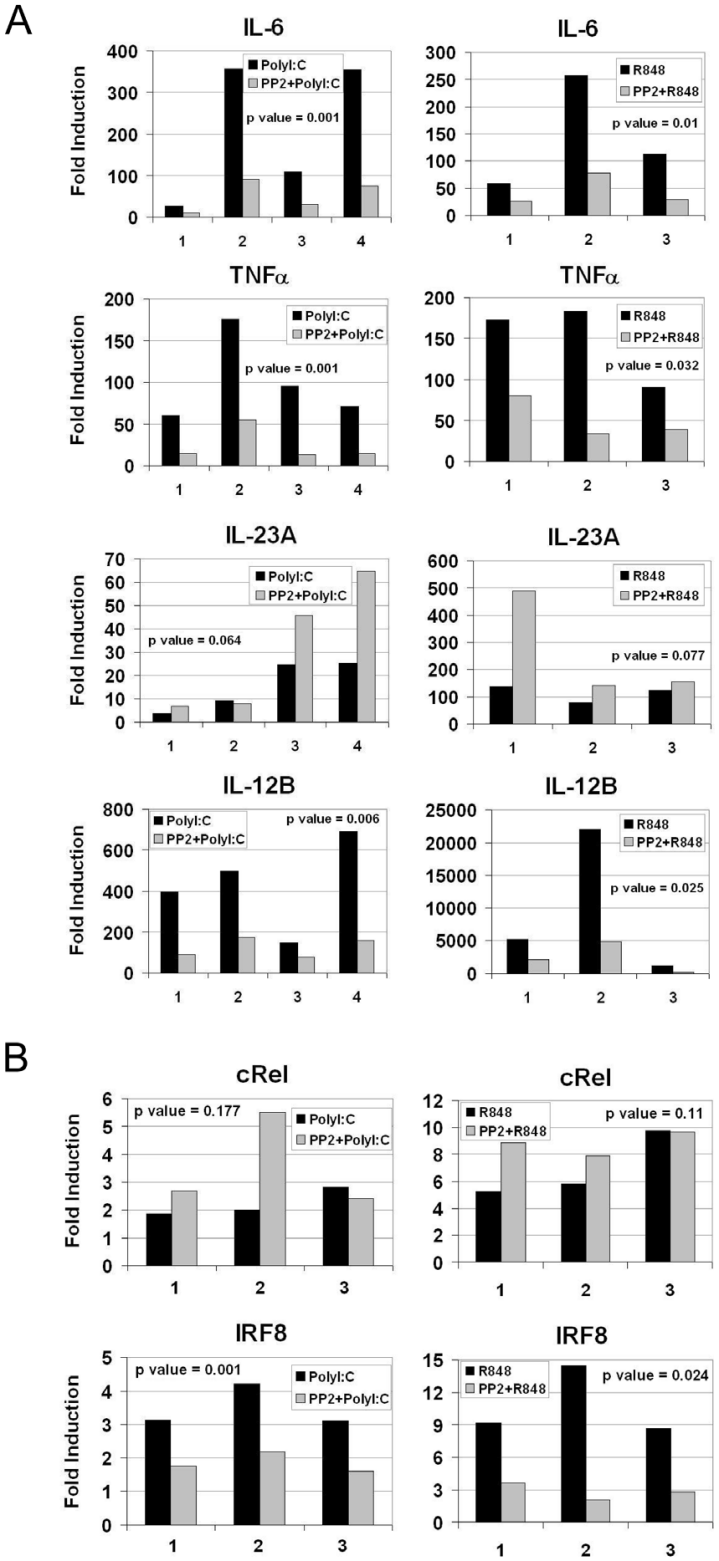
Src kinases inhibition does not affect expression of *IL-23A* and *cREL*. MoDC were pretreated or not with PP2 (20 µM) and stimulated with PolyI∶C (20 µg/ml) or R848 (10 µM) for 4 hours. qRT-PCR was performed to analyze expression of cytokine genes (A) or transcription factor genes (B). Fold induction for the expression of each gene compared to unstimulated cells is plotted. Expression levels for each analyzed gene were normalized to β-*Actin*. Three or four independent experiments and the p values for the differences between the groups are shown (p value <0.05 is significant).

To validate microarray data and to better investigate the relationship between IL-12 and IL-23 subunits, we performed a qRT-PCR on MoDC pretreated or not with PP2 and stimulated for 4 hours with either PolyI∶C or R848. These experiments confirmed microarray data showing that transcription of *IL-6, TNF*α and *IL-12B* was dramatically impaired in PP2-treated cells after 4 hours of stimulation with PolyI∶C or with R848 as demonstrated by data from four (with PolyI∶C) or three (with R848) independent experiments showing a statistical significant difference (p<0,05) between the two experimental groups (Figure 4A). On the contrary, the induction of *IL-23A* was not inhibited but most of the time enhanced upon Src kinases inhibition by PP2 (Figure 4A), although there was no statistical significant difference (p>0,05) between the two experimental groups. In order to identify the mechanism responsible for the different effect of Src kinases inhibition in the expression of IL-12 and IL-23 subunits, we focused our attention on the factors that regulate the transcription of these cytokines. It is known that IRF8 is a key transcription factor for production of *IL-12, IL-6 and TNF*α [27], [28], while recent evidence shows that transcription of the *IL-23A* subunit is mainly regulated by the NF-κB family member, c-Rel [29], [30]. Indeed qRT-PCR data from three independent experiments showed that in both MoDC stimulated with PolyI∶C or R848, *c-Rel* transcription was not affected by treatment with PP2 (no statistical significant difference (p>0,05) between the two experimental groups was observed), while *IRF-8* up-regulation was impaired upon inhibition of Src kinases with a statistical significant difference (p<0,05) between the two experimental groups (Figure 4B). These data, which confirmed the microarray data (Table 1), suggest that Src kinases activity modulate IRF8 responsive genes induced by TLR agonists.

### Src kinases inhibition decreases the production of IL-12 in response to TLR agonists but does not affect the production of IL-23

**Figure 5:**
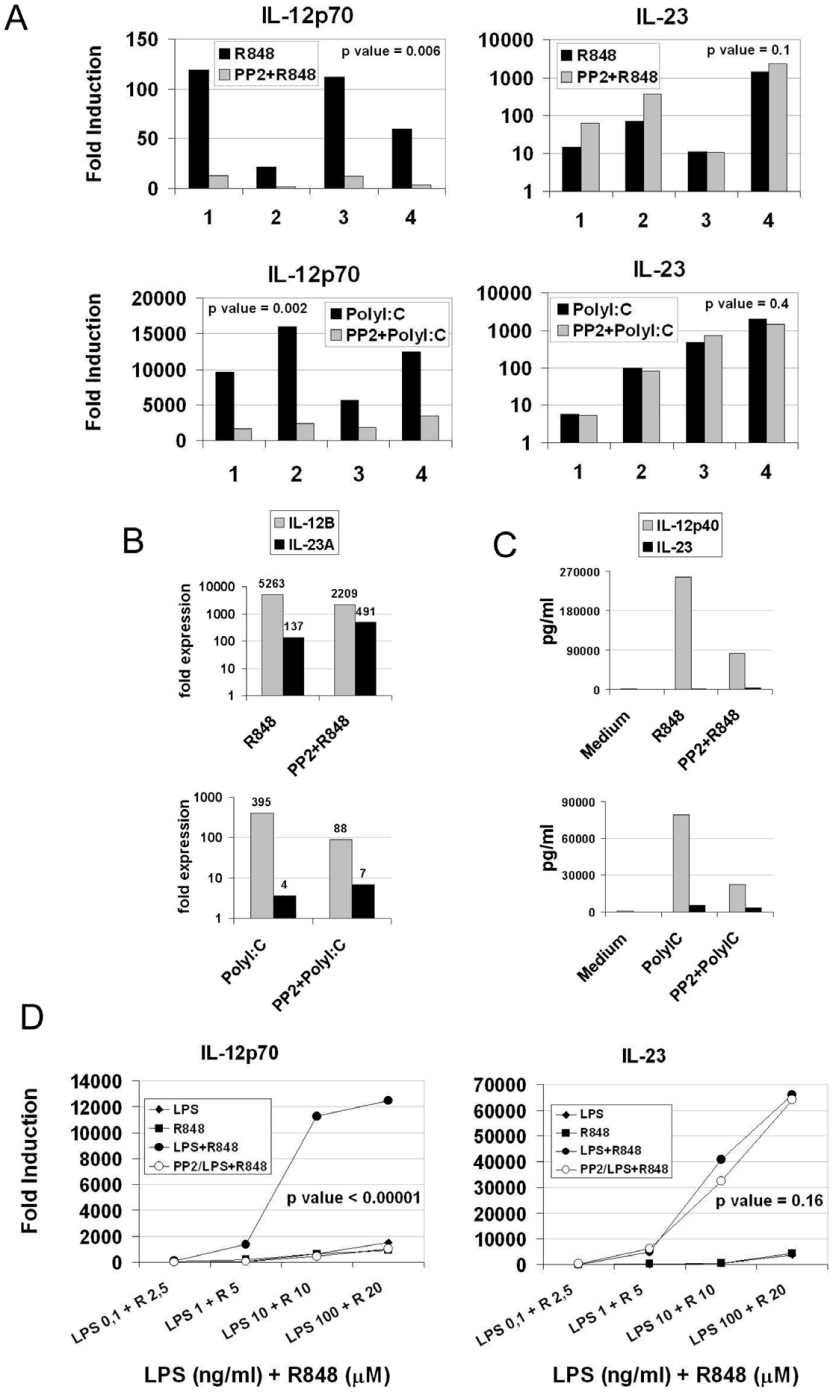
Src kinases inhibition results in imbalanced IL-12/IL-23 production. (A-C) MoDC were pretreated or not with PP2 (20 µM) and stimulated with PolyI∶C (20 µg/ml) or R848 (10 µM). (A) IL-12p70 and IL-23 release in the supernatant after 24 hours of stimulation was measured by ELISA. Fold induction for the levels of each cytokine compared to unstimulated cells is plotted. Data from four independent experiments and the p values for the differences between the groups are shown (p value <0.05 is significant). (B) qRT-PCR for IL-12B and IL-23A genes was performed after 4 hours of stimulation and expressed as fold increase over basal expression in unstimulated cells. Numbers indicates values of fold induction for each column. (C) IL-12p40 and IL-23 release in the supernatant after 24 hours of stimulation was measured by ELISA. Numbers indicates values of pg/ml for each column. (D) MoDC were pretreated or not with PP2 (20 µM) and stimulated with increasing combined doses of LPS and R848. IL-12p70 and IL-23 release in the supernatant after 24 hours of stimulation was measured by ELISA. Average fold induction of four independent experiments for the levels of each cytokine compared to unstimulated cells is plotted, and the p values for the differences between the groups are shown (p value <0.05 is significant).

The finding that *IL-23A* gene transcription is not inhibited upon treatment with PP2, while expression of its beta subunit is inhibited, raises the question of which is the effect of Src kinases on IL-23 protein production. We tested supernatants of DC after 24-hour of stimulation with either R848 or PolyI∶C for IL-12 and IL-23 presence. We observed that stimulation of TLR3 and TLR8 results in striking differences in the secretion of IL-12 and IL-23: PolyI∶C leads to a higher secretion of IL-12p70, while R848 preferentially induces IL-23 production (Figure 5A). As already observed in Figure 1A, we found that PP2 was able to impair IL-12 production, but unexpectedly did not affect IL-23 production following both TLR stimulations as shown by data from four independent experiments and their statistical analysis (Figure 5A). This differential effect of PP2 on the production of IL-12p70 and IL-23 was observed also at lower concentrations of inhibitor (Figure S4).

This result appears in conflict with data showing impaired up-regulation of the IL-12B mRNA, which should result in a decreased production of both IL-12 and IL-23. However, a direct comparison of IL12B and IL23A mRNA levels measured by qRT-PCR revealed that even in PP2 treated cells the mRNA levels of IL12B are higher compared to IL23A (Figure 5B). This observation suggests that in the assembling of the IL-23 the p40 subunit is in excess compared to the p19 subunit, while the latter is likely the limiting component. Indeed, a great excess of IL-12p40 compared to total IL-23 could be detected by ELISA in the supernatants of stimulated MoDC, even after Src kinases inhibition (Figure 5C). On a molar base the excess of IL-12p40 varied from 322 to 30 fold after R848 stimulation, and from 26 to 9 after PolyI∶C stimulation, in the absence and in the presence of PP2, respectively. This model, which indicates that p19 is the only limiting subunit for the assembly of IL-23, accounts for the absence of any decrease in the levels of total IL-23 cytokine following treatment with PP2. It was then critical to analyze what is the effect of Src kinases inhibition on the expression of IL-12A (p35) subunit of active IL-12 upon TLR agonist stimulation in MoDC. qRT-PCR analysis showed that PP2 was able to impair IL-12A up-regulation in R848-stimulated cells (Figure S5A) but not in PolyI∶C stimulated cells. However, a direct comparison of IL-12B and IL-12A mRNA levels suggests that while reduction of IL-12p70 levels in R848-stimulated cells reflects the inhibition of both subunits, in PolyI∶C stimulated cells IL-12A is in excess and IL-12B is very likely the limiting subunit (Figure S5B), thus influencing the protein levels of active IL-12p70. Unfortunately, this hypothesis could not be tested by an absolute measurement of monomeric IL-12A protein levels, since no-reagent for the detection of this subunit is available.

Dendritic cells express different TLRs and thus can be simultaneously activated by different PAMPs. Indeed, it was reported that a concurrent engagement of TLR8 with either TLR4 or TLR3 results in a synergistic effect on cytokine production [31]. We investigated if Src kinases can modulate cytokine production in MoDC even after simultaneous TLR activation. Cytokines were measured following MoDC stimulation with increasing concentrations of R848 and LPS, alone or simultaneously. As previously reported, a remarkable enhancement in the production of TNFα (data not shown), IL-12 and IL-23 (Figure 5D) was observed when both stimuli were provided. The synergistic effect on IL-12 production was completely abolished by inhibition of Src kinases, while no effect was observed on the synergistic production of the IL-23 cytokine, showing that activation of Src kinases is required for a balanced production of inflammatory cytokines in DC upon stimulation by multiple TLR agonists. IL-27 is a dimeric cytokine member of the IL-12 family and it has been described as a negative regulator of Th17 subset development [32]. It is constituted by a p28 and EBI3 subunits, paralogs of IL-12A and IL-12B, respectively. Because of its homology with IL-12, it was relevant to investigate the effect of PP2 on this cytokine. Indeed, we found that IL-27 production was not affected by Src kinases inhibition neither in PolyI∶C nor in R848-stimulated cells (Figure S6).

### Src kinases inhibition compromises Th1 differentiation but does not impair the ability of MoDC to promote the survival of Th17 cells

**Figure 6:**
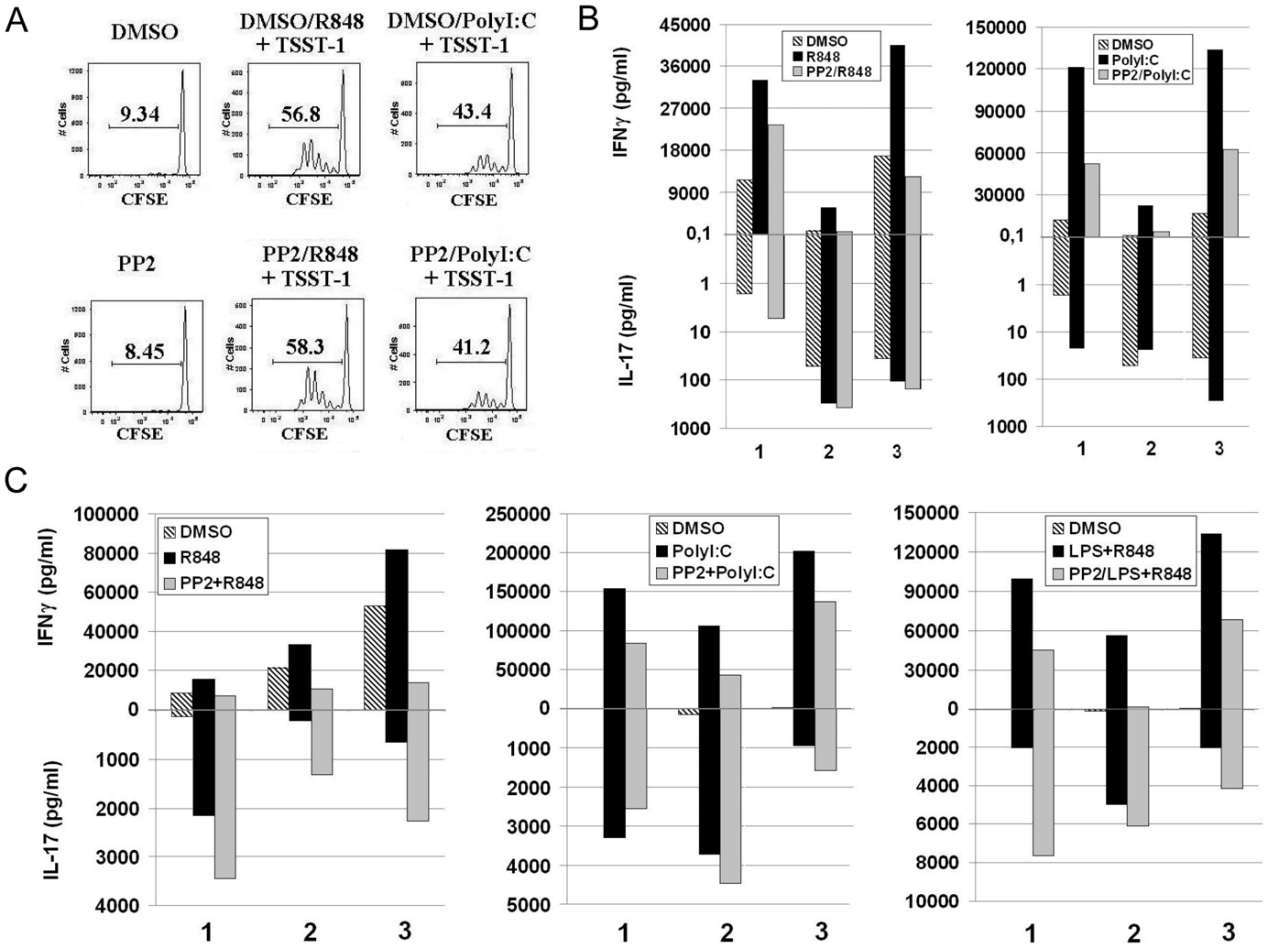
Imbalanced IL-12/IL-23 production results in a functional effect on T cells activation. (A) MoDC were pretreated or not with PP2 (20 µM), stimulated with PolyI∶C (20 µg/ml) or R848 (10 µM) for 6 hours, washed extensively and cultured with CFSE-labeled heterologous naïve CD4+ T cells in the presence of the superantigen TSST-1 (25 pg/ml). (B–C) MoDC were pretreated or not with PP2 (20 µM), stimulated with PolyI∶C (20 µg/ml), R848 (10 µM) or LPS (100 ng/ml)+R848 for 6 hours, washed extensively and cultured in fresh medium for 48 hours. After 48 hours supernatants were collected and added to naïve CD4+ T cells (B) or total CD4+ T cells (C), in the presence of anti-CD3 and anti-CD28 antibodies. After 7 days of incubation, T cells were restimulated with anti-CD3 and anti-CD28 and incubated for 7 additional days. At day 14, supernatants were collected and IL-17 and IFNγ were detected by ELISA or Mesoscale assays, respectively. Data from three independent experiments are shown.

Our data clearly show that Src kinases play an important role in cytokine production by MoDC upon stimulation with different TLRs. However, inhibition of Src kinases in these cells has a different impact on the production of IL-12 or IL-23. These two cytokines exert different, if not opposite functions during an immune response. While the bioactive form of IL-12, IL-12p70, is critical for differentiation of naïve T cells towards a Th1 cell type [33], [34], IL-23 is responsible for maintenance and survival of Th17 cells. We investigated if the imbalanced production of cytokines caused by Src kinases inhibition, translates into a functional effect on differentiation and maintenance of different T helper cell subsets. To asses the effect of Src kinases inhibition on the ability of MoDC to induce proliferation of naïve T cells, we co-cultured PolyI∶C- and R848-stimulated MoDC, which were previously pre-treated or not with PP2, with allogeneic naïve T cells for 4 days, in the presence of the superantigen TSST-1. We found that proliferation of naïve T cells was not affected by treatment of MoDC with PP2 (Figure 6A). This result is in agreement with the finding that induction of co-stimulatory molecules on DC did not require Src kinase activation (Figure 1B). The effect of Src kinase inhibition on the capacity of MoDC to shape polarization of naïve T cells was then evaluated. Naïve T cells were incubated for 7 days with MoDC supernatants in the presence of anti-CD3/CD28 antibodies, and then re-stimulated for other 7 days with anti-CD3/CD28 to expand polarized T cells. At day 14 supernatants from these T cells cultures were tested for IFNγ and IL-17. While supernatants from DC stimulated with R848 increased the levels of IFNγ production in naïve T cells - indicating a Th1 shift - we detected an impairment of this differentiation in T cell cultures containing supernatants from DC stimulated after inhibition of Src kinases. Impairment of Th1 cell differentiation was associated to an increase in IL-17 production by naïve T cells (Figure 6B). In case of supernatants from PolyI∶C-stimulated DC, they were able to induce a big Th1 polarization that was blunted when DC were inhibited by PP2 (Figure 6B). However no IL-17 was detected in naïve T cells differentiated in the presence of supernatants from PolyI∶C-stimulated DC when Src kinases were inhibited. This discrepancy between the results obtained with the two stimuli can be explained by the fact that in R848-stimulated cells the IL-12/IL-23 balance is shifted towards IL-23 production and this could be an important factor for the differentiation into Th17 cells. On the contrary, in PolyI∶C stimulated cells, the low levels of IL-23 compared to IL-12p70 are probably not sufficient to promote a Th17 shift of naïve CD4 T cells in vitro and that other factors are required, as previously showed by other studies [9]–[12]. To asses the effect of Src kinases inhibition on the capacity of MoDC to sustain the differentiation and survival of pre-polarized T helper cells, we stimulated total CD4+ T cells with anti-CD3 and anti-CD28 for 7 days in the presence of supernatants from MoDC which were stimulated by a single TLR agonist or a combination of TLR stimulators in the presence or not of Src kinase inhibition. CD4+ T cells were then re-stimulated with anti-CD3/CD28 for 7 additional days, and supernatants collected at day 14 were tested for IFNγ and IL-17 (Figure 6C). Supernatants form stimulated MoDC induced IFNγ production by CD4+ T cells in all cases (PolyI∶C, R848 and LPS+R848 stimulation of DC) but IFNγ levels were dramatically reduced when MoDC were pretreated with PP2. This reduction was associated to an increase in IL-17 production, likely indicating a better survival of the Th17 subset compared to the Th1 subset (Figure 6C). This shift occurred despite the presence of IL-27, a negative regulator of Th17 cells, in the supernatants of DC. Indeed, we found that IL-27 production was not affected by Src kinases inhibition neither in PolyI∶C nor in R848-stimulated cells (Figure S6). This finding is in agreement with a well established role of IL-23 in survival of memory Th17 cells. We also tested if the homing capacity of CD4+ T cells incubated with supernatants of DC was affected by inhibition of Src kinases. For this purpose we analyzed the surface expression of CCR6, CCR5, CCR9 and CXCR3 on CD4+ T cells that were stimulated as in Figure 6C. However, there was no significant difference in the expression of all four chemokine receptors on CD4+T cells incubated with supernatants from PolyI∶C or R848 stimulated DC in the presence and in the absence of PP2 (data not shown), suggesting that the homing capacity of T cells was not affected by Src kinases inhibition in MoDC.

## Discussion

In this paper we discuss the role of Src family kinases in cytokine production by human DC upon stimulation with TLR agonists. Cytokine production is a major feature of DC maturation and it is critical in triggering T cell activation. We previously showed that activation of Src kinases is a key step in TLR4 signaling in MoDC. Inhibition of Src kinases indeed resulted in a block of inflammatory cytokine release resulting in a clear impairment of Th1 differentiation [25]. TLR4 signaling is characterized by both MyD88- and TRIF-dependent signaling cascades. In this study, in order to dissect the role of Src kinases activation in the TRIF- and MyD88-dependent pathways we investigated the effects of Src kinases inhibitor PP2 in MoDC stimulated with TLR3 or TLR8 agonists. We demonstrated that following TLR3 and TLR8 activation, Src kinases control the activation of c-Jun and IRF1, while have no effect on NF-κB and ERK signaling cascade. Additionally, the microarray analysis presented here, confirmed by the qRT-PCR and secreted cytokines analyses, showed that Src kinases inhibition had a similar effect on the transcriptional changes induced by TLR3 and TLR8 activation excluding a pathway-specific involvement of these kinases in the TRIF or MyD88 signaling cascades. In particular, we found that Src kinases modulate the expression of several cytokines including IL12, TNFα and IL6. As previously observed for TLR4, inhibition of Src kinases had no effect on the induction of a maturation phenotype upon stimulation by PolyI∶C or R848, resulting in DC still able to induce naïve T cell proliferation. These data show that Src kinases control only some specialized aspects of the DC maturation process and that these kinases have a central role in the induction of most of the inflammatory cytokines, but not in the production of IL-23.

IL-23 is an inflammatory cytokine produced by mature DC, which was shown to support survival and full effector functions of Th17 cells, a new identified T helper population characterized by the production of IL-17 [13]. These cells have been implicated in the pathogenesis of autoimmune diseases [8], [35], [36]. Interestingly, IL-23 shares high homology with IL-12, the key cytokine responsible for Th1 differentiation as the IL-12p40 subunit (IL-12B) can either be associated to IL-12p35 (IL-12A) or to IL-23p19 (IL-23A), forming the bioactive form of IL-12 or IL-23, respectively. We show that Src kinases control the induction of the IL-12p40 subunit, but have no role in the transcription and production of the IL-23A subunit. Thus, inhibition of Src kinases results in a severe imbalance in the IL-12/IL-23 axis, revealing a peculiar role for Src kinases in cytokine production. In agreement with the cytokine expression data we observed that Src kinases inhibition affects the up-regulation of the transcription factor IRF8, which controls the expression of inflammatory cytokines such as IL-12 and TNFα, bus does not impair the induction of cRel, which regulates the expression of the IL-23A subunit.

IL-12 and IL-23 have different effects on T helper cell differentiation and survival. It was therefore of paramount importance to establish if the observed IL-12/IL-23 imbalance would result in a functional effect on the type of immune response. We showed that a variation in the IL-12/IL-23 ratio has consequences on the polarization or survival of T helper cells. MoDC in which IL-12p70 production was impaired due to Src kinases inhibition are less efficient in inducing differentiation of naïve T cells into Th1 cells. This is in agreement with a role for IL-12 as a Th1-polarizing cytokine. Many groups have showed that Th17 cells can be induced from naïve T cells by culturing these cells with a cocktail of inflammatory cytokines such as IL-1β, IL-6 and TGFβ, and that IL-23 is necessary but not sufficient for the differentiation of Th17 cells [9]–[12]. As we have shown that Src kinases inhibition results in impairment not only of IL-12 but also of IL-6, IL-1β and TNFα production, we would have expected that under this condition we could not induce detectable levels of IL-17-producing T cells from naïve precursors. Never the less, supernatants of R848-stimulated DC were able to trigger IL-17 production from naïve T cells, likely because of the high levels of IL-23 induced in comparison to other stimuli, and IL-17 was further increased when src kinases were inhibited in MoDC. On the other hand, Oppman et al showed that the main biological effects of IL-23 can be observed on survival of memory Th17 cells and not on naïve T cells [13]. We show that co-culturing of total T cells with MoDC supernatants resulted in maintenance and survival of Th17 cells. The increase in IL-17 production was associated to a dramatic decrease in IFNγ release, suggesting that Src kinases inhibition can produce a shift from Th1 to Th17 cells. Th17 cell subset has been suggested to be a major player in the pathogenesis of autoimmune diseases. This hypothesis is based on a series of studies on both knockout mice and human samples from patients affected by autoimmune diseases. However, a recent study claims that Th17 cells could have an anti-inflammatory role in *H.Pylori*-induced gastritis in mice [17]. Another study has also lately challenged the notion about a pathogenic role of Th17 cells in autoimmune inflammation. Flavell and coworkers showed that IL-17 has a protective function in a mouse model of inflammatory bowel disease by down-regulation of Th1 specific gene expression and a modulation of T cell mediated colon inflammation [37]. Thus, the role of Th17 in inflammation and autoimmune diseases has not yet been definitively elucidated. Our findings showing that the pharmacological modulation of DC maturation by Src inhibitors can inhibit Th1 responses without affecting Th17 differentiation could be used as the basis of further investigations aimed at exploiting the potential use of these drugs for the treatment of inflammatory diseases.

## Materials and Methods

### Donors

Buffy coats from healthy donors were obtained from the Blood Transfusion Section, Alta Val D'Elsa Hospital (Poggibonsi, Italy). Informed consent was obtained before all blood donations. The study protocol was approved by the Novartis Research Center ethical committee and conforms to the ethical guidelines of the 1975 Declaration of Helsinki.

### Cell culture and reagents

PBMC were isolated from buffy coats of healthy donors using Ficoll gradient. Monocytes were isolated from PBMC with anti-CD14 magnetic beads (Miltenyi Biotec) and cultured with human recombinant GM-CSF (50 ng/ml) (Gentaur) and human recombinant IL-4 (5 ng/ml) (Gentaur) for 5–7 days. Differentiation of monocytes to Dendritic cells was routinely assessed by Flow cytometry detection of CD14 and CD1a surface molecules: only DC populations that showed no expression of CD14 and an expression of CD1a higher than 70% were used for the experiments. The culture medium was RPMI 1640 (GIBCO) supplemented with 2 mM L-glutamine, 1% sodium pyruvate, 1% nonessential amino acids, 100 g/ml kanamycin (GIBCO), 5×10^−5^ M 2-mercaptoethanol (Sigma) and 10% heat-inactivated fetal calf serum (Hyclone). Naïve T cells and total CD4+ T cells were isolated from PBMCs by negative selection using magnetic beads based kits from Miltenyi Biotec. T cells were cultured in IMDM (GIBCO) supplemented with Penicillin-Streptomycin-Glutamine (GIBCO), and 10% heat-inactivated fetal calf serum (Hyclone). PP2 was purchased from Alexis and added to MoDC used 20 min before stimulation with TLR agonists. PolyI∶C was from Invivogen, while LPS and R848 were purchased from Alexis. For T cell proliferation, naive CD4^+^ T cells were labeled with CFSE (Invitrogen) at 0,5 µM. Polyclonal T cells stimulation was induced with anti-CD3 and anti-CD28 (from BD Biosciences) at 1 µg/ml. TSST-1 was purchased from Sigma Aldrich.

### Flow cytometry

Flow cytometry was performed on FacsCanto or FACS LSRII instruments using DIVA software (Becton Dickinson) and data were analyzed using Flowjo software (Treestar Inc.). Proliferation of naïve T cells was evaluated after measuring dilution of CFSE fluorescence. MoDC phenotypic maturation was evaluated by extracellular staining with anti-CD83-FITC and anti-CD86-APC (BD Biosciences).

### Cytokine detection

Mesoscale Assay Human-Proinflammatory 7-spot, Human IFNβ single-spot and Human TH1/TH2 7-spot kits (MSD Technology) were used for detection of inflammatory cytokines and of polarizing cytokines, respectively, following manufacturer's instructions. Sandwich ELISA using matched paired antibodies was performed for detection of IL-23, IL-12p40, IL-12p70, IL-17A (eBioscience) and IL-27 (R&D Systems).

### Western blot analysis

Cells were lysed in lysis buffer [Tris 50 mM (pH 7.5), NaCl 300 mM, and Triton X-100 0.5%] supplemented with proteases and phosphatase inhibitors. Protein concentration in the postnuclear lysates was measured by BCA Protein Assay (Pierce) and equal amounts of protein lysates (60 µg) were loaded on 10% SDS-PAGE. Gels were transferred on nitrocellulose using iBlot Dry Blotting System (Invitrogen). Filters were blocked with 5% dry skimmed milk and blotted with the following specific primary antibodies: mouse monoclonal antibody to phosphorylated c-Jun (Ser 63) (KM1) (Santa Cruz Biotechnologies) and to c-Src (Upstate); rabbit polyclonal antibodies to c-Jun, IRF1 (Santa Cruz Biotechnologies), phosphorylated c-Src, ERK1/2 and phosphorylated ERK1/2 (Thr 201/Tyr 204) (Cell Signaling); antiserum to actin (Sigma); rabbit antiserum to IκB (a gift of Dr Antonio Leonardi, University of Naples, Italy). Blots were then incubated with the appropriate HRP-conjugated secondary antiserum (Jackson Immunoresearch), and revealed with the WestPico chemiluminescence system (Pierce). Filters were stripped for 10 min with ReBlot Plus Strong Antibody Stripping Solution (Millipore).

### Microarray analysis and quantitative real-time PCR

Total RNA from human MoDC was extracted with Rneasy Mini Kit (Qiagen, Hilden, Germany). For quantitative real-time-PCR (qRT-PCR) experiments cDNA was synthesized using the *SuperScript III First-Strand Synthesis System for RT-PCR* (Invitrogen). qRT-PCR was performed using iQ SYBRGreen SuperMix (Biorad). For the microarray analysis total RNA was amplified using the Agilent Low RNA Input Linear Amplification kit. In a first experiment RNA obtained from MoDC from three independent donors stimulated with PolyI∶C (20 µg/ml) or R848 (10 µM) for 4 hours were labeled using Cy5 and co-hybridized with RNA from unstimulated MoDC labelled with Cy3. In a second set of experiments total RNA from MoDC from the same donors stimulated with PolyI∶C (20 µg/ml) or R848 (10 µM) in the presence of PP2 (20 µM) was labelled with Cy3 and co-hybridized with Cy-5 labeled RNA from cells stimulated by the same amount of PolyI∶C or R848 in the absence of PP2. In all experiments 400 ng of total RNA was used as starting material. The efficiency of incorporation of the Cy5 or Cy3 dye was measured by NanoDrop analysis. Equal amounts of labelled Cy5 and Cy3 cRNAs were co-hybridized onto the Agilent Technologies 44K whole human genome microarray, detecting over 40,000 transcripts, for 17 hours at 65°C following the Agilent protocol. Raw images were first analyzed by using the GenePix 6.0 software (Molecular Devices), and the data were then transferred to the BASE 1.2 database/analysis software. For each spot, local background was subtracted from the mean fluorescence intensity of the Cy3 and Cy5 dyes. Spot intensities were then normalized by the global mean. Spots with a signal-to-noise ratio 4 in both channels were filtered. The average intensity ratio of repeated spots from three experimental replicates was estimated by geometric mean. The accuracy and statistical significance of the observed ratios were determined using the Student's t-test. The complete set of microarray data has been submitted to the Array Express database EMBL-EBI (http://www.ebi.ac.uk/arrayexpress/) with accession number E-TABM-1018.

### Statistical analysis

One tail paired Student's t-test was performed to evaluate statistically significant differences between two groups. A p value <0.05 was considered statistically significant.

## Supporting Information

Table S1 (0.11 MB XLS)

Table S2 (0.11 MB XLS)

Figure S1 c-Src kinase is activated upon stimulation with R848 and PolyI:C. Human MoDC were pretreated or not with PP2 (20 µM) and stimulated with PolyI∶C (20 µg/ml) or R848 (10 µM) for the indicated time. Phosphorylated (Y418) c-Src was detected by WB on total cell lysates. After stripping filter was re-blotted with an antibody directed against total c-Src.
(1.06 MB TIF)

Figure S2 Src kinases inhibition results in impaired IFNβ production. Human MoDC were pretreated or not with PP2 (20 µM) and stimulated with PolyI∶C (20 µg/ml). After 24 hours IFNβ released in the supernatants was detected using a Mesoscale assay. Fold induction for the levels of IFN-beta compared to unstimulated cells is plotted. Three independent experiments and the p values for differences between the groups are shown (p value <0.05 is significant).
(0.63 MB TIF)

Figure S3 Src kinases are required for accumulation of c-Jun and IRF1. Human MoDC were pretreated or not with PP2 (20 µM) and stimulated with PolyI∶C (20 µg/ml) or R848 (10 µM) for the indicated time. IκB, phospho-ERK, phospho-cJun and IRF1 were detected by WB on total cell lysates. After stripping filters were re-blotted with antibodies to actin, total ERK, total cJun and actin, respectively. (A) Intensity of the bands in Figure 2 of the manuscript was quantified by Image J and represented as fold induction over samples from unstimulated cells. (B) Densiometric analysis of WB detection for p-cJun, cJun, IRF1 and IκB from three independent experiments. For each experiment fold induction over samples from unstimulated cells were normalized to actin expression and plotted. The p values for differences between the groups are shown (p value <0.05 is significant).
(1.50 MB TIF)

Figure S4 IL-12p70 production is inhibited even at low concentrations of PP2. Human MoDC were pretreated with the indicated doses of PP2 for 20 minutes at 37°C, and then stimulated with PolyI∶C (20 µg/ml) or R848 (10 µM). After 24 hours supernatants were collected and IL-12p70 and IL-23 were measured by Mesoscale or ELISA, respectively.
(1.11 MB TIF)

Figure S5 Comparison between expression levels of IL-12 subunits. (A) MoDC were pretreated or not with PP2 (20 µM) and stimulated with PolyI∶C (20 µg/ml) or R848 (10 uM). qRT-PCR for IL-12A gene was performed after 4 hours of stimulation and expressed as fold increase over basal expression in unstimulated cells. (B) A comparison between IL-12A and IL-12B mRNA levels. Numbers on each column indicate values of fold induction. Data are representative of at least three experiments.
(1.13 MB TIF)

Figure S6 Src kinases inhibition does not affect IL-27 production. Human MoDC were pretreated or not with PP2 (20 µM) and stimulated with PolyI∶C (20 µg/ml) or R848 (10 µM). IL-27 released in the supernatants was detected after 24 hours of stimulation, by ELISA. Fold induction of IL-27 compared to unstimulated cells is plotted. Three independent experiments and the p values for differences between the groups are shown (p value <0.05 is significant).
(0.88 MB TIF)
