# Supplementary figures and images for: Src Kinases Are Required for a Balanced Production of IL-12/IL-23 in Human Dendritic Cells Activated by Toll-Like Receptor Agonists
Source: PLoS One. 2010 Jul 9;5(7):e11491. doi: 10.1371/journal.pone.0011491 (PMC2901334; doi:10.1371/journal.pone.0011491)

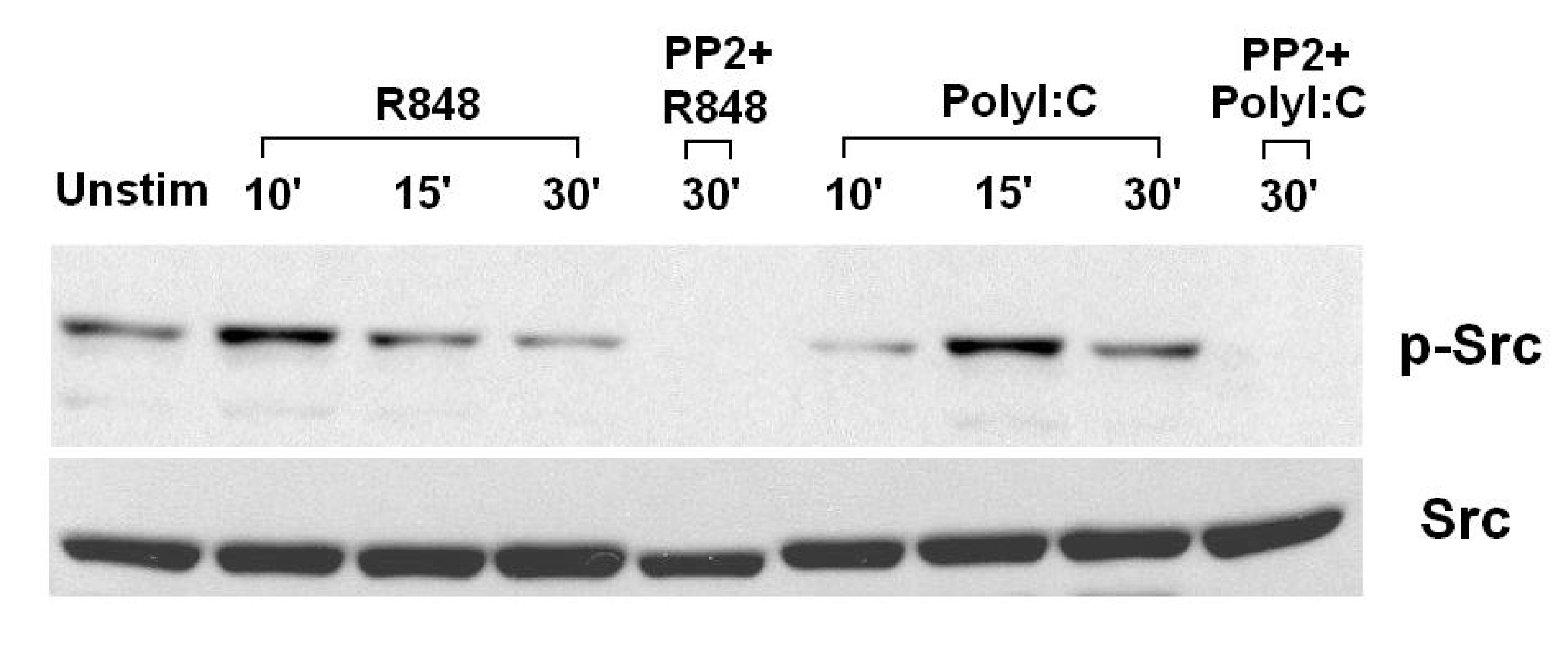

Supplement: Figure S1 — c-Src kinase is activated upon stimulation with R848 and PolyI:C. Human MoDC were pretreated or not with PP2 (20 µM) and stimulated with PolyI∶C (20 µg/ml) or R848 (10 µM) for the indicated time. Phosphorylated (Y418) c-Src was detected by WB on total cell lysates. After stripping filter was re-blotted with an antibody directed against total c-Src. (1.06 MB TIF) [file pone.0011491.s003.tif]

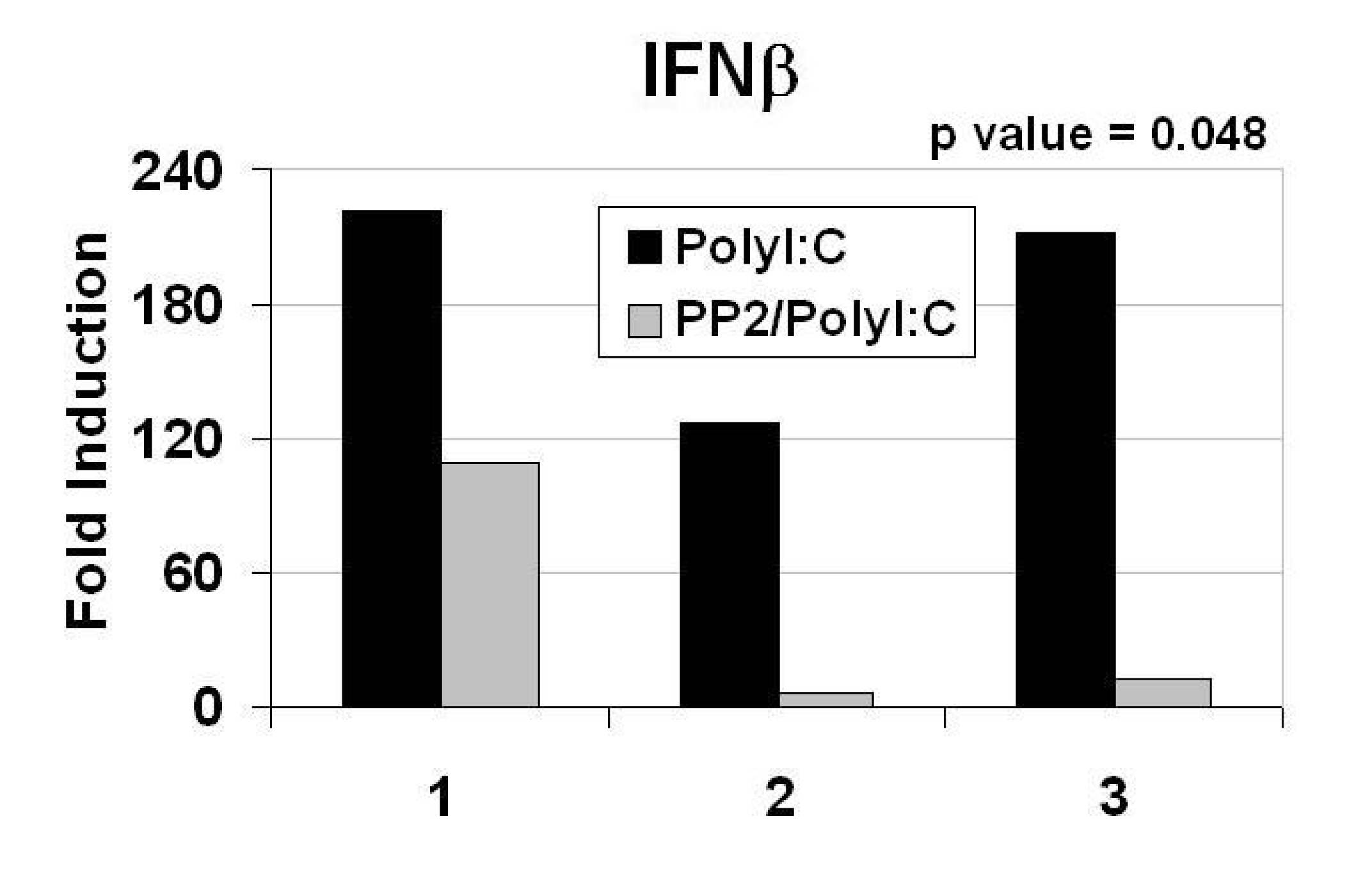

Supplement: Figure S2 — Src kinases inhibition results in impaired IFNβ production. Human MoDC were pretreated or not with PP2 (20 µM) and stimulated with PolyI∶C (20 µg/ml). After 24 hours IFNβ released in the supernatants was detected using a Mesoscale assay. Fold induction for the levels of IFN-beta compared to unstimulated cells is plotted. Three independent experiments and the p values for differences between the groups are shown (p value <0.05 is significant). (0.63 MB TIF) [file pone.0011491.s004.tif]

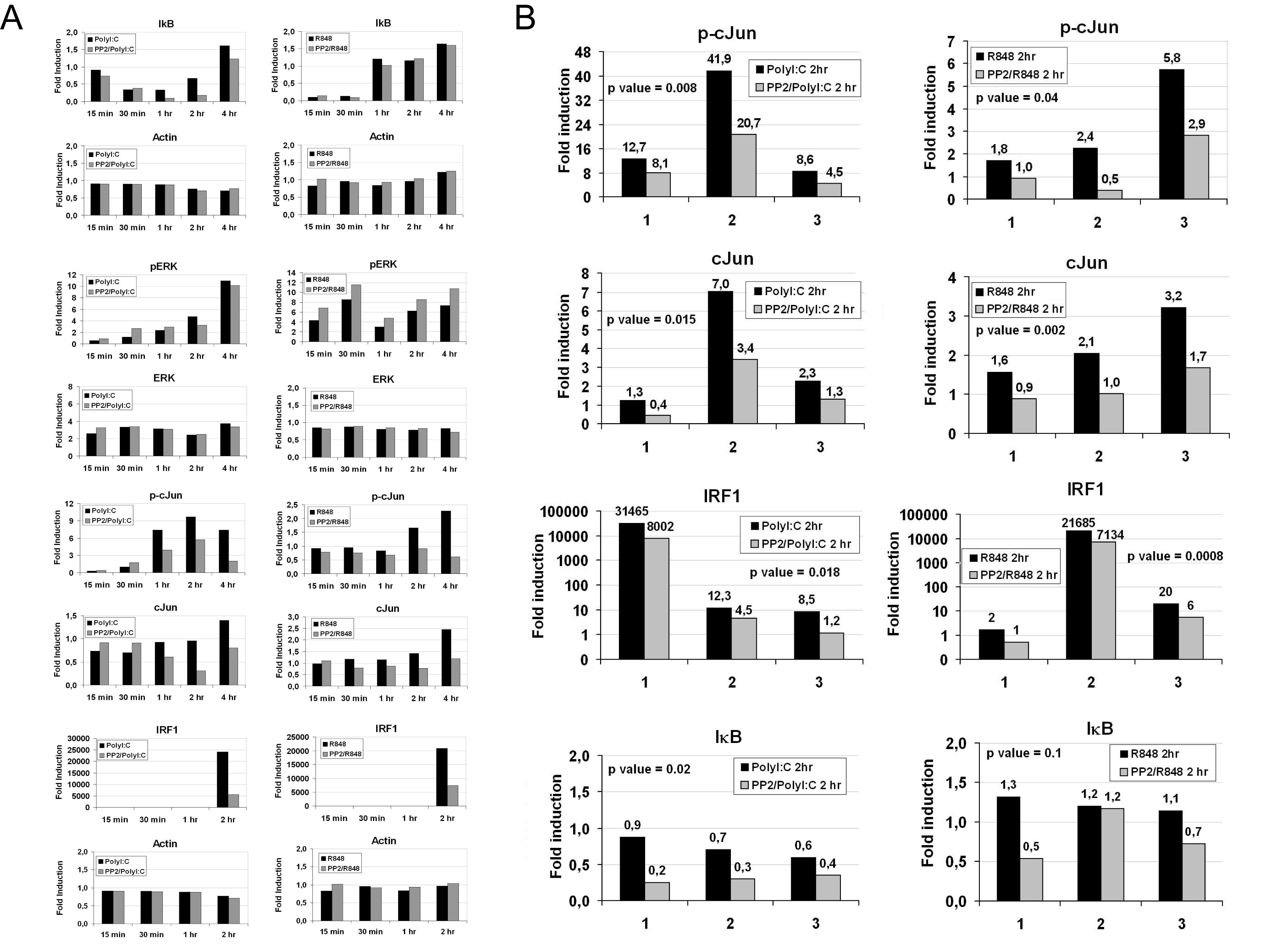

Supplement: Figure S3 — Src kinases are required for accumulation of c-Jun and IRF1. Human MoDC were pretreated or not with PP2 (20 µM) and stimulated with PolyI∶C (20 µg/ml) or R848 (10 µM) for the indicated time. IκB, phospho-ERK, phospho-cJun and IRF1 were detected by WB on total cell lysates. After stripping filters were re-blotted with antibodies to actin, total ERK, total cJun and actin, respectively. (A) Intensity of the bands in Figure 2 of the manuscript was quantified by Image J and represented as fold induction over samples from unstimulated cells. (B) Densiometric analysis of WB detection for p-cJun, cJun, IRF1 and IκB from three independent experiments. For each experiment fold induction over samples from unstimulated cells were normalized to actin expression and plotted. The p values for differences between the groups are shown (p value <0.05 is significant). (1.50 MB TIF) [file pone.0011491.s005.tif]

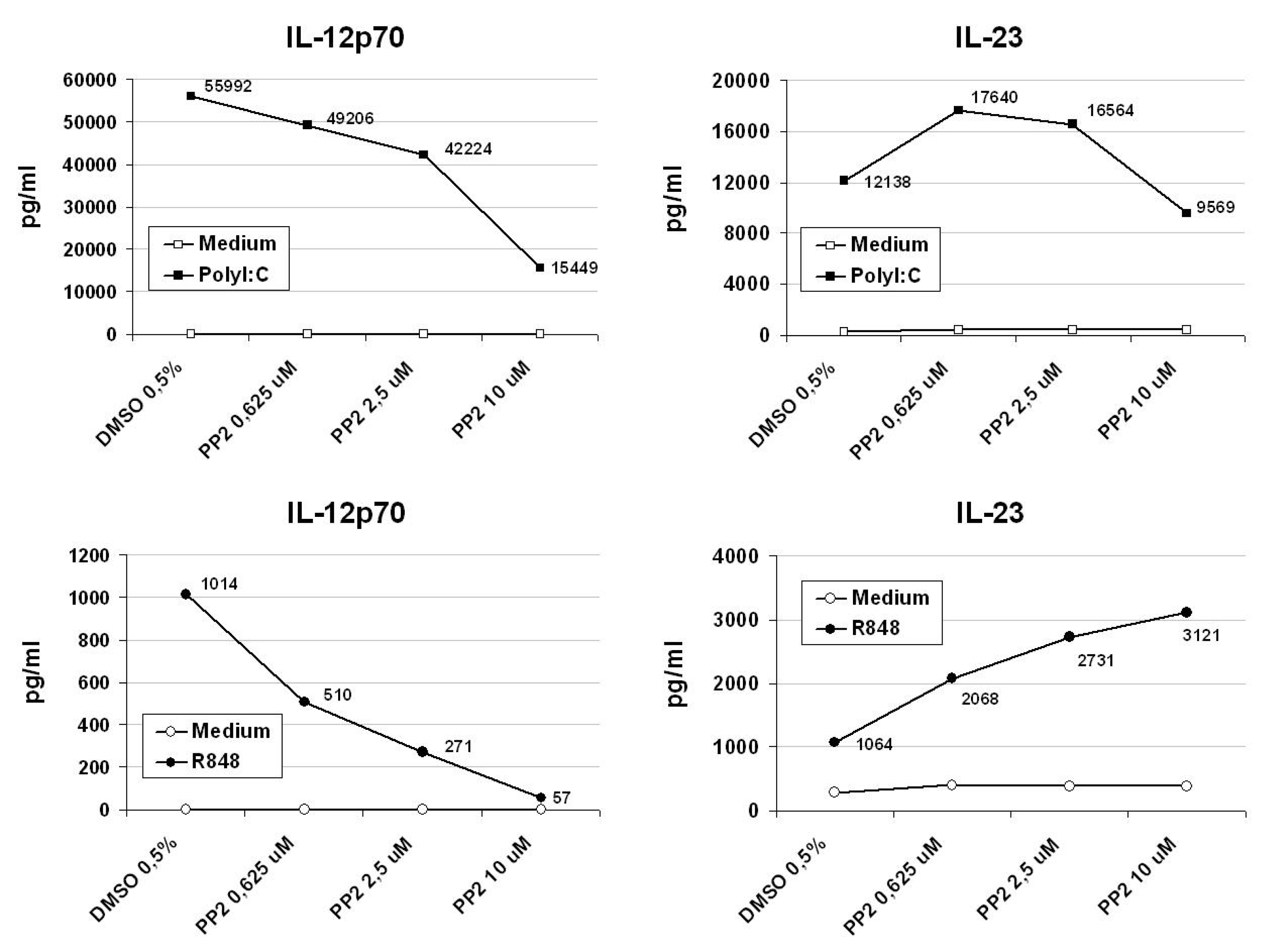

Supplement: Figure S4 — IL-12p70 production is inhibited even at low concentrations of PP2. Human MoDC were pretreated with the indicated doses of PP2 for 20 minutes at 37°C, and then stimulated with PolyI∶C (20 µg/ml) or R848 (10 µM). After 24 hours supernatants were collected and IL-12p70 and IL-23 were measured by Mesoscale or ELISA, respectively. (1.11 MB TIF) [file pone.0011491.s006.tif]

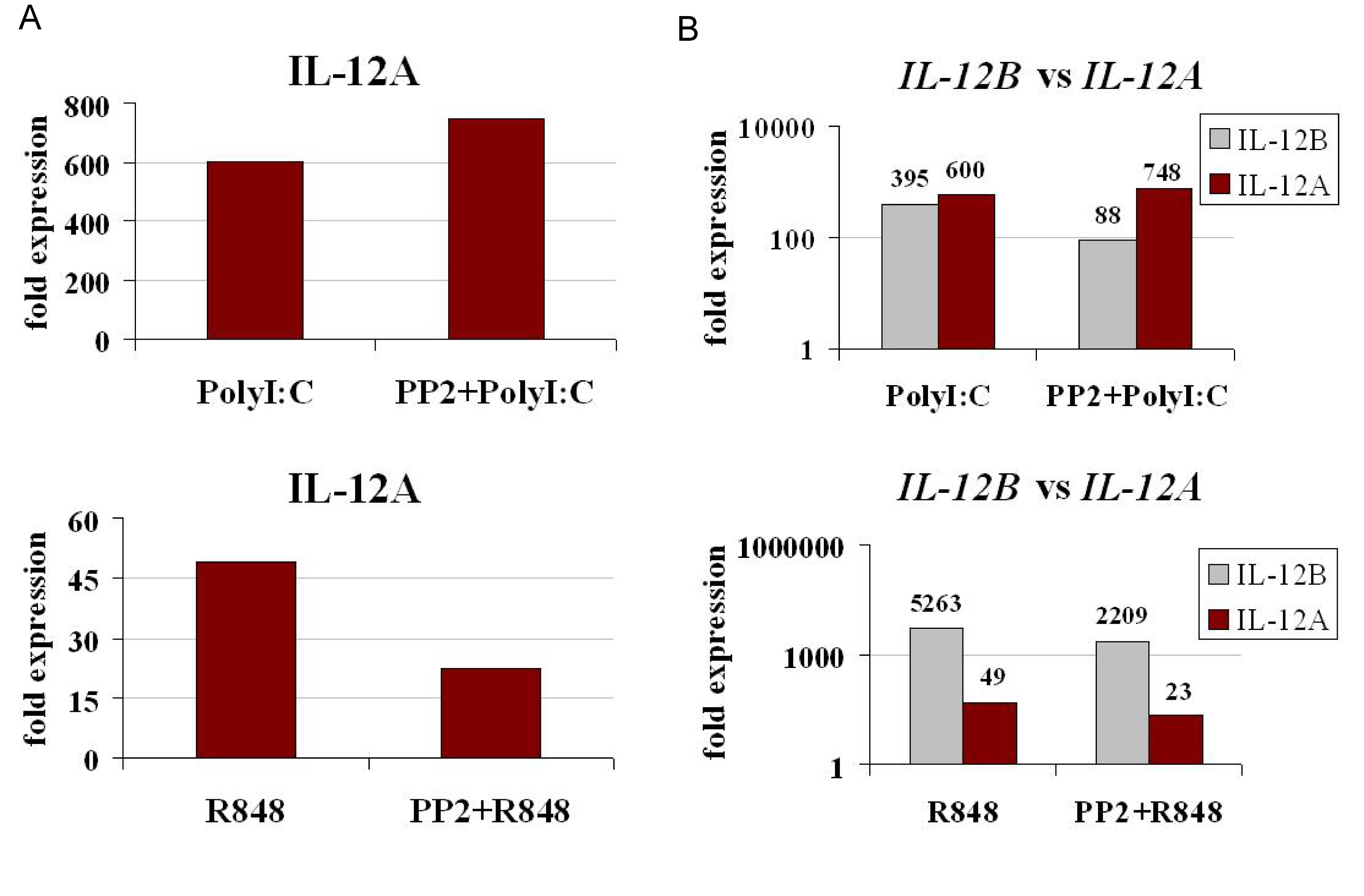

Supplement: Figure S5 — Comparison between expression levels of IL-12 subunits. (A) MoDC were pretreated or not with PP2 (20 µM) and stimulated with PolyI∶C (20 µg/ml) or R848 (10 uM). qRT-PCR for IL-12A gene was performed after 4 hours of stimulation and expressed as fold increase over basal expression in unstimulated cells. (B) A comparison between IL-12A and IL-12B mRNA levels. Numbers on each column indicate values of fold induction. Data are representative of at least three experiments. (1.13 MB TIF) [file pone.0011491.s007.tif]

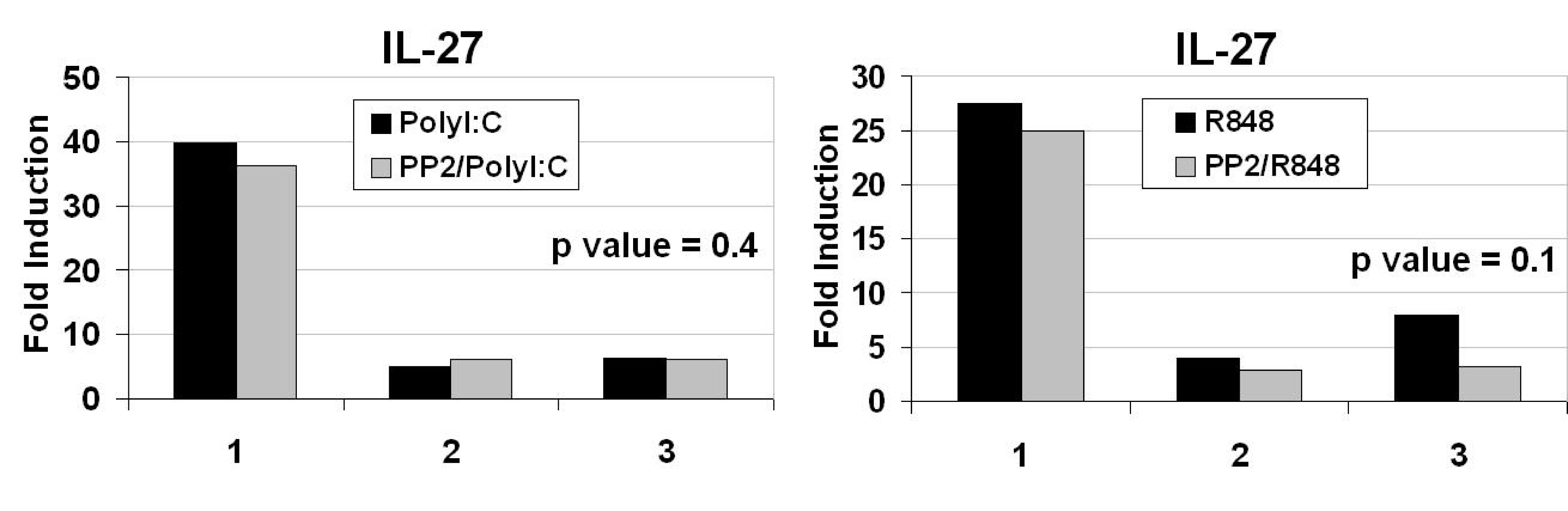

Supplement: Figure S6 — Src kinases inhibition does not affect IL-27 production. Human MoDC were pretreated or not with PP2 (20 µM) and stimulated with PolyI∶C (20 µg/ml) or R848 (10 µM). IL-27 released in the supernatants was detected after 24 hours of stimulation, by ELISA. Fold induction of IL-27 compared to unstimulated cells is plotted. Three independent experiments and the p values for differences between the groups are shown (p value <0.05 is significant). (0.88 MB TIF) [file pone.0011491.s008.tif]
